# Predicting Human Protein Subcellular Locations by Using a Combination of Network and Function Features

**DOI:** 10.3389/fgene.2021.783128

**Published:** 2021-11-05

**Authors:** Lei Chen, ZhanDong Li, Tao Zeng, Yu-Hang Zhang, ShiQi Zhang, Tao Huang, Yu-Dong Cai

**Affiliations:** ^1^ School of Life Sciences, Shanghai University, Shanghai, China; ^2^ College of Information Engineering, Shanghai Maritime University, Shanghai, China; ^3^ College of Food Engineering, Jilin Engineering Normal University, Changchun, China; ^4^ Bio-Med Big Data Center, CAS Key Laboratory of Computational Biology, Shanghai Institute of Nutrition and Health, University of Chinese Academy of Sciences, Chinese Academy of Sciences, Shanghai, China; ^5^ Channing Division of Network Medicine, Brigham and Women’s Hospital, Harvard Medical School, Boston, MA, United States; ^6^ Department of Biostatistics, University of Copenhagen, Copenhagen, Denmark; ^7^ CAS Key Laboratory of Tissue Microenvironment and Tumor, Shanghai Institute of Nutrition and Health, University of Chinese Academy of Sciences, Chinese Academy of Sciences, Shanghai, China

**Keywords:** protein subcellular location, protein-protein interaction network, GO enrichment, KEGG enrichment, feature selection, classification algorithm

## Abstract

Given the limitation of technologies, the subcellular localizations of proteins are difficult to identify. Predicting the subcellular localization and the intercellular distribution patterns of proteins in accordance with their specific biological roles, including validated functions, relationships with other proteins, and even their specific sequence characteristics, is necessary. The computational prediction of protein subcellular localizations can be performed on the basis of the sequence and the functional characteristics. In this study, the protein–protein interaction network, functional annotation of proteins and a group of direct proteins with known subcellular localization were used to construct models. To build efficient models, several powerful machine learning algorithms, including two feature selection methods, four classification algorithms, were employed. Some key proteins and functional terms were discovered, which may provide important contributions for determining protein subcellular locations. Furthermore, some quantitative rules were established to identify the potential subcellular localizations of proteins. As the first prediction model that uses direct protein annotation information (i.e., functional features) and STRING-based protein–protein interaction network (i.e., network features), our computational model can help promote the development of predictive technologies on subcellular localizations and provide a new approach for exploring the protein subcellular localization patterns and their potential biological importance.

## 1 Introduction

Eukaryotic organisms, such as human beings, have complicated cell structures with delicate functional membrane structures surrounded by effective compartments (Thul et al., 2017; Tjondro et al., 2019). The complicated membrane structures in eukaryotic cells have generally divided the intercellular space into the cytoplasm and the nucleus through the nuclear membrane (Yeagle, 1989; Mangeat et al., 1999). Specific organelles, such as the mitochondria, have a specific and independent membrane system (Set et al., 2019). The major components of these structures divide the intercellular space into different isolated rooms for independent biological reactions and interactions and restrict the intercellular localizations of proteins (Thul et al., 2017). For instance, the replication of DNA depends on various effective proteins and enzymes. However, some proteins, such as DNA polymerase and DNA ligase, are not synthesized in the nucleus, in which they function (Ganai and Johansson, 2016). Some proteins play a specific role in biological processes in the nucleus (Ganai and Johansson, 2016). Therefore, the subcellular localization controls the protein to some extent to act at the proper localization.

Given the limitation of technologies, the subcellular localizations of proteins are difficult to identify. Therefore, predicting the subcellular localization and the intercellular distribution patterns of proteins in accordance with their specific biological roles, including validated functions, relationships with other proteins, and even their specific sequence characteristics, is necessary. The computational prediction of protein subcellular localizations can be performed on the basis of the sequence and the functional characteristics. Sequence characteristics-based methods can be further divided into three kinds, namely, the N-terminal sorting method, amino acid composition-based prediction, and homology. The N-terminal sorting method is based on subcellular localization prediction. In 2006, researchers from Greece reported a subcellular localization predictor by using the N-terminal signaling sequence of the protein, resulting in a cross-validated accuracy of 87.1% in animals (Petsalaki et al., 2006). The amino acid composition of proteins is easy to determine and describe, but the models that use amino acid composition do not have good prediction performance. Therefore, amino acid compositions are generally used to accompany other characteristics, such as N-terminal sorting and homology. The homology considers another important feature subgroups of sequence characteristics. Predictors, such as the Proteome Analyst (Szafron et al., 2004) and the PairProSVM (Mak et al., 2008), have been validated to have a good performance for protein subcellular localization prediction. Recently, some advanced computational methods, such as deep learning, multiple kernel learning, etc. are adopted to learn features derived from protein sequence and set up prediction models (Wei et al., 2018; Ding et al., 2020).

Apart from the above sequence-based prediction methods, predicting the subcellular localization of proteins by using the functional annotation and correlations between proteins has attracted attention due to the accomplishment of human protein function annotation and the establishment of the protein–protein interaction (PPI) network. However, the extraction of protein functional features is quite difficult compared with extracting protein sequencing features. With the development of bioinformatics, the most widely used approaches have been established on the annotation and clustering of the gene ontology (GO) (Consortium, 2015) and the Kyoto Encyclopedia of Genes and Genomes (KEGG) pathways (Zhang and Wiemann, 2009). In these methods, the GO and the KEGG pathway terms are applied to describe and cluster proteins as optimal protein characteristics. GO has terms on cellular components that describe the general subcellular localization. Some predictors, such as the ProLoc-GO (Huang et al., 2008), the ILoc-Virus (Xiao et al., 2011), and the Cell-PLoc (Chou and Shen, 2008), combine the general description with the sequence characteristics, thereby establishing a novel and effective prediction method on subcellular localization. However, the functional annotation of proteins remains imperfect, and potentially new functions of proteins emerge. Therefore, additional methods should be presented to supplement current research.

In this study, the comprehensive PPI network provided by STRING (Szklarczyk et al., 2016) and GO/KEGG pathway annotations on proteins were employed to analyze the current proteins with known subcellular localizations. Qualitative and quantitative predictive models were established to identify the potential subcellular localizations of proteins on the basis of several machine learning algorithms, such as feature selection methods, classification algorithms. In addition to models, we also obtained some key proteins and functional terms that may provide important contributions for determining protein subcellular locations. As the first prediction model that used direct protein annotation information (i.e., functional features) and the STRING-based PPI network (i.e., network features), our computational model can help promote the development of predictive technologies on subcellular localizations and provide a new approach for exploring the protein subcellular localization patterns and their potential biological importance.

## 2 Materials and Methods

### 2.1 Data

The data used in this study were extracted from the Swiss-Prot (http://cn.expasy.org/, release 54.0) by searching the proteins annotated with “subcellular location”. Initially, 53,427 protein sequences were downloaded. Proteins with length shorter than 50 amino acids (e.g., protein fragments) and those with length longer than 5,000 amino acids (e.g., protein complexes) were excluded. Proteins containing unknown amino acid abbreviation, such as X, were also excluded. Protein sequences with high degree of similarity were also removed using the program CD-HIT (Li and Godzik, 2006) and a cutoff value of 0.7. Finally, only human proteins were studied. Thus, 4,986 protein sequences remained after these exclusions and were classified into 16 categories (Table 1).

**TABLE 1:** Number of proteins in each category.

Index	Category	Number of proteins
Class 1	Biological membrane	1,487
Class 2	Cell periphery	35
Class 3	Cytoplasm	506
Class 4	Cytoplasmic vesicle	70
Class 5	Endoplasmic reticulum	190
Class 6	Endosome	25
Class 7	Extracellular space or cell surface	649
Class 8	Flagellum or cilium	3
Class 9	Golgi apparatus	98
Class 10	Microtubule cytoskeleton	48
Class 11	Mitochondrion	345
Class 12	Nuclear periphery	33
Class 13	Nucleolus	112
Class 14	Nucleus	1,285
Class 15	Peroxisome	46
Class 16	Vacuole	54

### 2.2 Feature Representation

Good representation of proteins is very important to build efficient models for identification of human protein subcellular locations. In this study, each protein was represented by three groups of features, where one group was derived from PPI network, two groups were extracted from functional terms (GO and KEGG pathway). Their descriptions are as follows.

#### 2.2.1 Network Features Derived From PPI Network

The initial PPI network was downloaded from STRING (version 9.0) (Szklarczyk et al., 2011) (http://string.embl.de/), which contained known and predicted protein interaction. The interaction network considers proteins as its nodes and has an edge between two proteins if they can interact with each other. Furthermore, each edge was assigned a weight, which was defined as the confidence score of the corresponding interaction. As such score was obtained by considering several aspects of proteins, it can widely measure the associations of proteins. Given a protein, a feature vector was constructed, where each component indicated a protein in the PPI network. Each component was defined as the confidence score of the interaction between the protein and the corresponding protein of such component. If such interaction did not exist, the component was set to zero. For an easy description, these features were called network features. As there were 20,770 proteins in the PPI network, 20,770 network features were generated for each protein.

#### 2.2.2 Functional Features Derived From KEGG Pathway

The immediate neighborhood method is usually used for predicting the function of a query protein on the basis of the other proteins with known functions (Sharan et al., 2007). A query protein interacts with many neighboring proteins in the STRING network (Szklarczyk et al., 2011). With these neighboring proteins, we can assess the relationship between the query protein and one KEGG pathway. Let the neighboring proteins and the query protein constitute a protein set *PS*. For a KEGG pathway, proteins in such pathway comprised another protein set, denoted by *KP*. The relationship between the query protein and the KEGG pathway, called KEGG enrichment score, was defined as the −log10 of the hypergeometric test *p* value (Carmona-Saez et al., 2007; Cai et al., 2010) on above-constructed protein sets. All obtained enrichment scores on all KEGG pathways were collected in a vector, comprising the functional KEGG features of the protein. 297 KEGG pathways were considered, inducing 297 functional KEGG features.

#### 2.2.3 Functional Features Derived From GO

Similarly, the relationship between the query protein and one GO term can be obtained. For a GO term, let *GP* be a protein set consisting of proteins annotated by such GO term. The relationship was defined as the −log10 of the hypergeometric test *p* value (Cai et al., 2010; Li et al., 2012) on *PS* and *GP*. The obtained value was called GO enrichment score. Likewise, GO enrichment scores on all GO terms were collected in a vector, constituting the functional GO features of the query protein. 20,681 GO terms were involved, generating 20,681 functional GO features.

### 2.3 Boruta Feature Filtering

The Boruta feature filtering (Kursa and Rudnicki, 2010) can screen features that are relevant to target sample labels on the basis of the random forest (RF) in a wrapper manner. The Boruta feature filtering iteratively identifies key features by comparing the importance scores that correspond to the real and the shuffled features. The Boruta approach has three steps: 1) copying the training data and shuffling the feature values for new shuffled data to be produced; 2) training the RF classifier on the produced shuffled data and calculating the importance score for each feature; and 3) evaluating the importance score of each feature in the original training data and removing the real features with remarkably lower importance scores than the shuffled features. By executing the above steps with a few iterations, Boruta approach selects the relevant features.

This study adopted the Boruta program retrieved from https://github.com/scikitlearn-contrib/boruta_py. For convenience, it was performed with its default parameters.

### 2.4 Minimum Redundancy Maximum Relevance

The mRMR (Peng et al., 2005) can select and rank informative features in accordance with the following assumptions. On the one hand, the mRMR selects features with minimum redundancy among themselves. On the other hand, the mRMR selects features with maximum relevance with class labels. Therefore, the mRMR only selects the features that satisfy minimum redundancy and maximum relevance simultaneously by using mutual information. These features are important in distinguishing the class labels for follow-up classification modeling. In fact, two feature lists can be obtained through the mRMR method. The MaxRel feature list ranks features based on their relevance to class labels, whereas the mRMR feature list sorts features by further considering the redundancies among features. Evidently, from the mRMR feature list, we can obtain a compact feature subspace for a given classification algorithm. Thus, this study only adopted the mRMR feature list.

The present study used the mRMR program downloaded from http://home.penglab.com/proj/mRMR/. Likewise, default parameters were adopted to execute such program.

### 2.5 Incremental Feature Selection

IFS, an ordered feature selection approach (Liu and Setiono, 1998), can determine the best number of selected features in an iteration manner. The IFS first constructs a series of feature subsets from the ranked features supplied by a feature ranking (e.g., mRMR feature list). For instance, the first feature subset consists of the top 10 features, and the second feature subset consists of the top 20 features, and so on. Next, the IFS trains a model on the training samples, which consist of features from each feature subset, based on a given classification algorithm. Such classification model performance is evaluated by 10-fold cross-validation (Kohavi, 1995). Finally, the model with the highest performance is found out, which was called the optimum model. The feature subset used in this model was called the optimum feature subset.

### 2.6 Classification Algorithm

To execute the IFS method, one classification algorithm is necessary. This study tried four classification algorithms: 1) RF (Breiman, 2001), 2) Support vector machine (SVM) (Cortes and Vapnik, 1995), 3) k-nearest neighbor (kNN) (Cover and Hart, 1967), 4) Decision tree (DT) (Swain and Hauska, 1977). These algorithms have been widely used to tackle various biological problems (Jia et al., 2020; Zhou et al., 2020; Chen et al., 2021; Pan et al., 2021; Yang and Chen, 2021; Zhang et al., 2021a; Zhang et al., 2021b; c).

#### 2.6.1 Random Forest

RF builds an assemble classification algorithm depending on many tree classifiers. The predicted sample label/category of RF is determined using multiple tree classifiers by an aggregating vote. Notably, RF usually adopts the final consensus results in accordance with the average of all decision trees’ predictions, aiming to avoid overfitting and improve the performance robustness of learned models because a subtle difference among decision trees exists in RF. To quickly implement RF, the tool “RandomForest” in Weka (https://www.cs.waikato.ac.nz/ml/weka/) (Hall et al., 2009) was employed. Default parameters were used.

#### 2.6.2 Support Vector Machine

As a classification algorithm based on statistical learning theory, the SVM can map samples to a given category. The SVM transforms samples from a low-dimensional space to a high-dimensional space by using a kernel function (e.g., Gaussian kernel) and can divide samples of each label/category by maximizing the data interval in high-dimensional space. The SVM can further predict the test samples’ label/category in accordance with the interval to which this new sample belongs. In this study, we used the SVM optimized by the sequence minimization optimization (SMO) (Platt, 1998a; Platt, 1998b) algorithm. This type of SVM is implemented by the tool “SMO” in the Weka (https://www.cs.waikato.ac.nz/ml/weka/) (Hall et al., 2009).

#### 2.6.3 k-Nearest Neighbor

The kNN builds a classification model by using a voting scheme (Theilhaber et al., 2002; Zhang and Srihari, 2004; Yu et al., 2016). In the sample space, the class labels of the kNNs of a given sample were used to produce a predicted class label for a new sample. In the learning of kNN classification model, the nearest neighbors are selected from the training data, where *k* is a given parameter that usually ranges from 1 to 10. Briefly, the kNN includes several calculation steps: 1) calculating the distance between the test sample and all the training samples in the feature space; 2) ranking the training samples in accordance with their distance with the test sample; 3) selecting the *k* training samples with least distance to the test sample (i.e., *kNNs*); 4) determining the distribution of class labels of the *k* nearest training samples; and 5) using the class label with highest distribution frequency as the predicted class label for the test sample. The tool “IBK” in Weka (https://www.cs.waikato.ac.nz/ml/weka/) (Hall et al., 2009) implements the kNN algorithm, which was directly employed in this study.

#### 2.6.4 Decision Tree

The DT can produce interpretative rules that easily explain the classification and the regression models for wide applications in many research fields. In brief, DT is a nonparametric supervised learning method and uses a white box model with the IF-TEHN format to provide definite indications of individual features for classification and regression. A common construction strategy of DT is greedy algorithm, which achieves satisfactory performance with reasonable computational cost. The corresponding pack collected in Scikit-learn (https://scikit-learn.org/stable/) (Pedregosa et al., 2011), which implements an optimized version of the CART algorithm with the Gini index, was used to build DT model in this study.

### 2.7 Synthetic Minority Oversampling Technique

Table 1 shows that the analyzed data were unbalanced numbers of samples with different labels (i.e., different classified categories). Thus, the SMOTE (Chawla et al., 2002) was applied. It can produce new samples for the minor sample classes iteratively until the sample numbers of these minor sample classes are equivalent to that of the major sample class. The balanced data can improve the construction of the classification models. In this study, we used the tool “SMOTE” in the Weka (https://www.cs.waikato.ac.nz/ml/weka/) (Hall et al., 2009), which implements SMOTE method. Samples generated by SMOTE were not used in the methods of Boruta and mRMR because these newly added samples may influence the results of these two methods, which cannot fully reflect actual distribution of subcellular locations of proteins.

### 2.8 Performance Evaluation

In this study, the Matthew correlation coefficients (MCC) (Matthews, 1975) within 10-fold cross-validation (Kohavi, 1995) was used to evaluate the prediction performance of each classification model. MCC is a commonly used measurement and ranges between −1 and +1, achieving +1 when the classification model has the best performance. The multiclass version of MCC is proposed by Gorodkin (Gorodkin, 2004). Our analyzed data contained 16 categories, and MCC was calculated as follows:

$$MCC=\frac{\mathrm{cov}\left(X,Y\right)}{\sqrt{\mathrm{cov}\left(X,X\right)\mathrm{cov}\left(Y,Y\right)}}$$ (1)

where cov(⋅,⋅) represents the covariance of two matrices, *X* is a 0–1 matrix that indicates the predicted class of each sample, and *Y* is a 0–1 matrix that represents the actual classes of all samples.

Besides, the performance of each constructed model was also evaluated by other measurements, including individual accuracy on each category and overall accuracy.

## 3 Results

**FIGURE 1:**
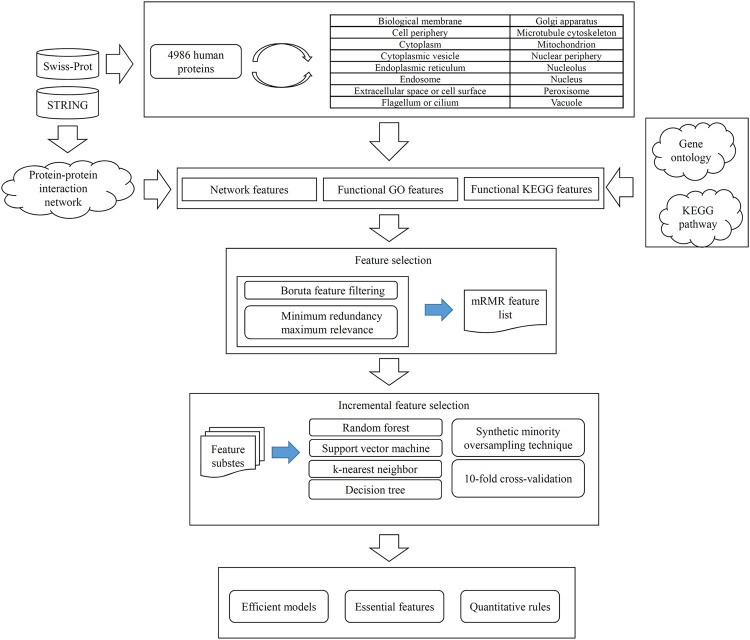
Entire procedures for constructing and evaluating protein subcellular location prediction models. Human proteins and their subcellular location information are retrieved from Swiss-Prot. Each protein is represented by three feature groups: network features, functional KEGG features, and functional GO features. All features are analyzed by Boruta and minimum redundancy maximum relevance one by one, resulting in an mRMR feature list. Such list is fed into the incremental feature selection method, incorporating four classification algorithms, synthetic minority oversampling technique and 10-fold cross-validation, to build efficient models, extract essential features and access quantitative rules.

In this study, we conducted a computational investigation on identification of human protein subcellular locations. The entire procedures are illustrated in Figure 1. Detailed results were described in this section.

### 3.1 Results of Boruta and Minimum Redundancy Maximum Relevance Methods

**FIGURE 2:**
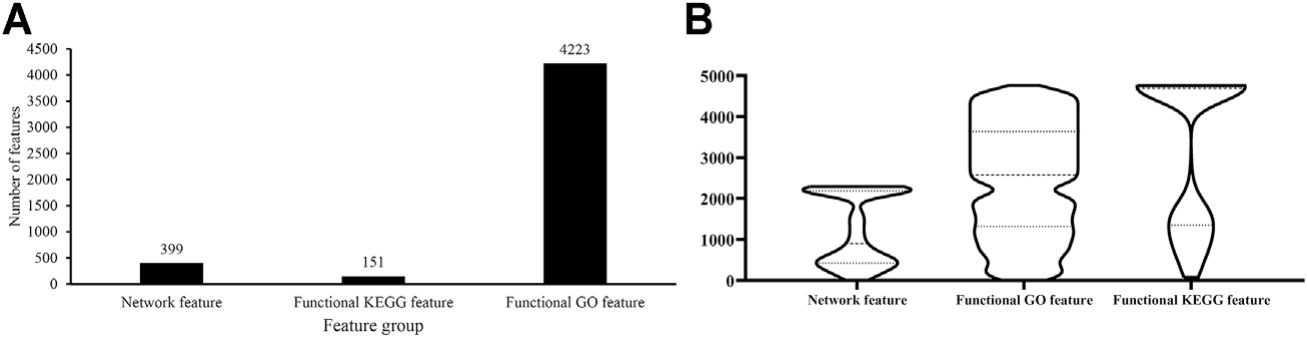
Analysis of features selected by Boruta. **(A)** Distribution of features selected by Boruta on three feature groups; **(B)** Violin plot to show ranks of features in three feature groups which are obtained by mRMR method.

As described in *Feature Representation*, each protein was represented by lots of network, functional KEGG and functional GO features. The Boruta method was first applied to analyze all features. Irrelevant features were discarded. 4,773 features remained, which are provided in Supplementary Table S1. Among these features, 399 were network features, 151 were functional KEGG features, and 4,223 were functional GO features, which are shown in Figure 2A. Evidently, functional GO features occupied most features (∼88%).

For these 4,773 features, the mRMR method followed to analyze their importance. An mRMR feature list was generated, as listed in Supplementary Table S1. We counted ranks of features in each feature group and plotted a boxplot in Figure 2B. It can be observed that network features received many high ranks in the mRMR feature list although their quantity was not most. This suggested that network features can provide key contributions for determination of protein subcellular locations.

### 3.2 Results of IFS Method

**FIGURE 3:**
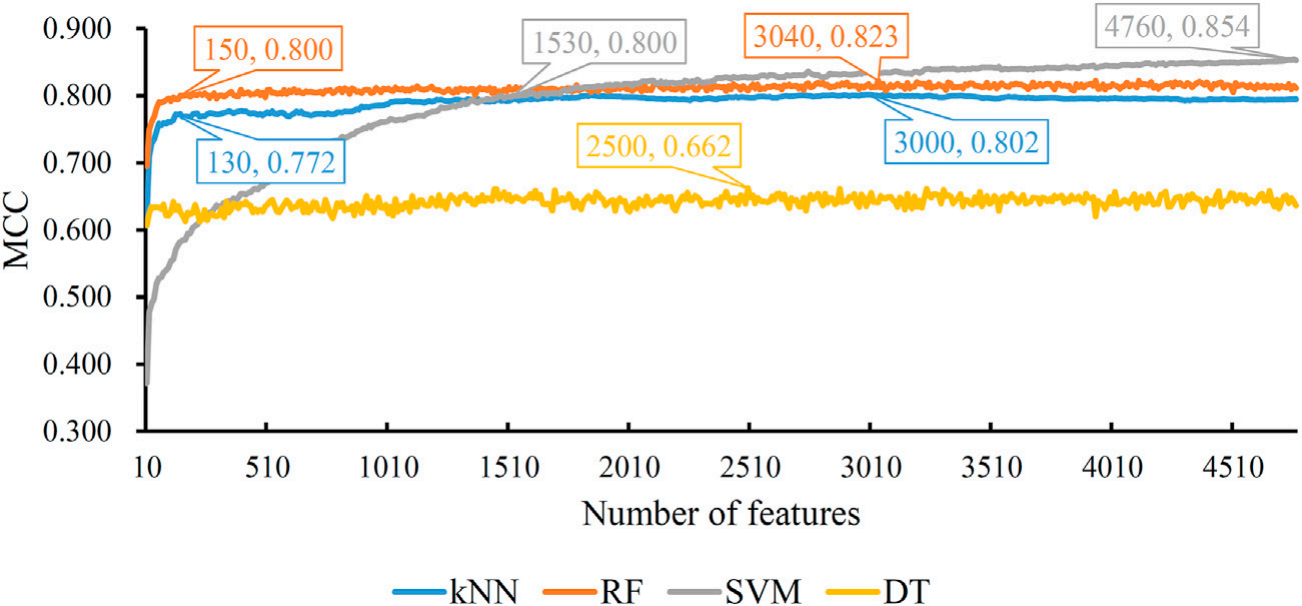
IFS with four classification algorithms on mRMR feature list of network and functional features. The highest MCC values obtained by four classifications are 0.802, 0.823, 0.854, and 0.662, respectively. kNN, RF, and SVM can yield quite good performance when much less features are adopted.

Based on the mRMR feature list, the IFS method was executed. 477 feature subsets were constructed with step 10. On each feature subset, a model was built based on each of the four classification algorithms (RF, kNN, SVM, and DT). The model was further evaluated by 10-fold cross-validation. The evaluation results, including MCC, overall accuracy and individual accuracies on 16 categories, for RF, kNN and SVM are listed in Supplementary Table S2. For an easy observation, an IFS curve was plotted for each classification algorithm, which is shown in Figure 3. For kNN, the highest MCC was 0.802, which was obtained by using top 3,000 features in the mRMR feature list. Thus, we can construct an optimum kNN model with these features. The overall accuracy of such model was 0.830 (Table 2). For RF, it produced the highest MCC of 0.823 when top 3,040 features were adopted, thereby building the optimum RF model with these features. The overall accuracy of such model was 0.852 (Table 2). As for SVM, the highest MCC was 0.854. This performance was obtained by using top 4,760 features in the list. Accordingly, an optimum SVM model was set up with these features. Its overall accuracy was 0.879 (Table 2). Evidently, each optimum model provided good performance with MCC higher than 0.800, suggesting combination of network and functional features can really capture the essential properties of proteins.

**TABLE 2:** Performance of key models for identification of human protein subcellular locations.

Classification algorithm	Number of features	Overall accuracy	MCC
k-nearest neighbor	3,000	0.830	0.802
	130	0.805	0.772
Random forest	3,040	0.852	0.823
	150	0.833	0.800
Support vector machine	4,760	0.879	0.854
	1,530	0.833	0.800
Decision tree	2,500	0.716	0.662

**FIGURE 4:**
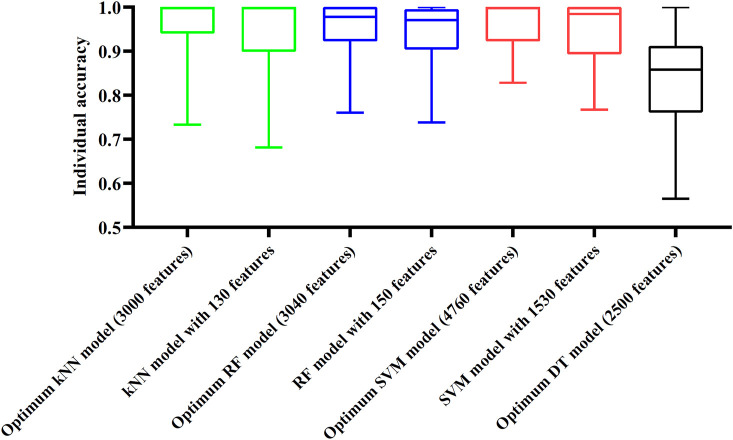
Box plot to show performance of some models on 16 categories. For three classification algorithms (kNN, RF, and SVM), models with much less features can provide similar performance to the optimum models. Optimum DT model yields much lower performance.

Although three optimum models were set up as mentioned above, their efficiencies were not very high because lots of features were used. To build models with high efficiency, we carefully checked the performance of three classification algorithms on different feature subsets. Other three models using much less features were constructed, where the kNN model used the top 130 features, RF model adopted the top 150 features and SVM model used the top 1,530 features (Figure 3). Although these models adopted much less features, their performance was only a little lower than those of the optimum models. This fact can be concluded from Table 2 and Figure 4. Thus, these models can be efficient tools for identification of protein subcellular locations.

For DT, we conducted the same IFS procedure. The IFS results are provided in Supplementary Table S3, which induced a curve, as shown in Figure 3. It can be observed that the highest MCC was 0.662 when top 2,500 features were adopted. Accordingly, we can set up an optimum DT model using these features. The overall accuracy was 0.716, as listed in Table 2. Evidently, such performance was much lower than that of the optimum kNN/RF/SVM model. It was also lower than those of the models with higher efficiency mentioned in the above paragraph. The individual accuracies on 16 categories yielded by this DT model were also obviously lower than those of other models, as shown in Figure 4. However, the utility of DT model was not to identify protein subcellular locations. Different from kNN, RF, and SVM, which were complete black-box algorithms, the classification procedures of DT were open. Thus, it can provide much more biological insights than other three classification algorithms.

### 3.3 Results of Quantitative Rules

**FIGURE 5:**
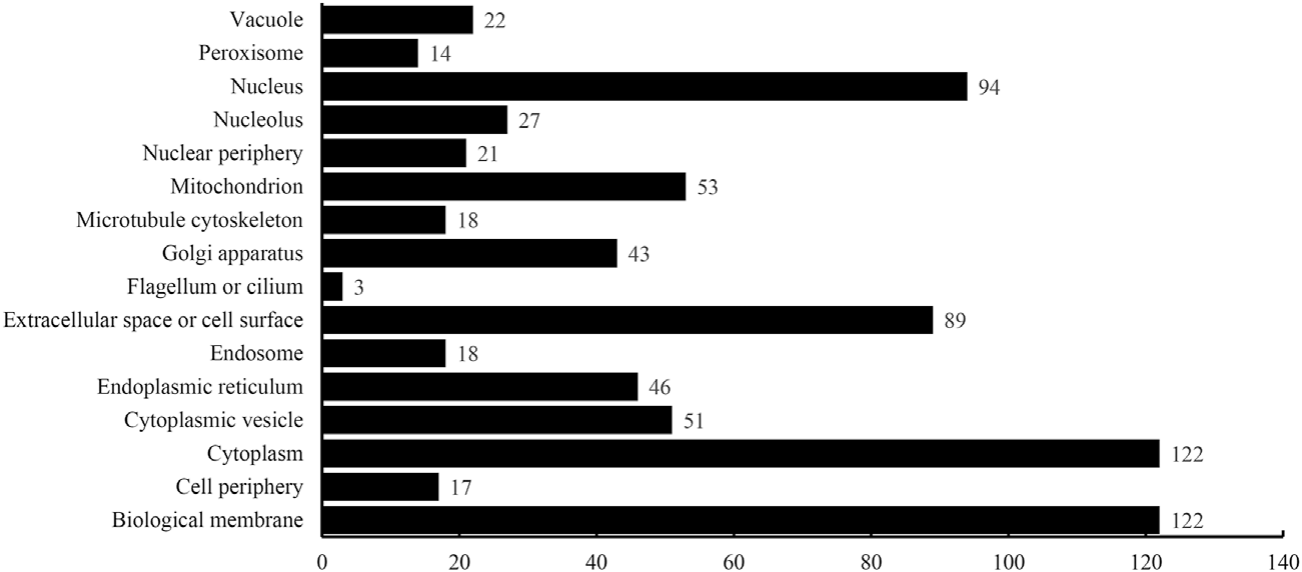
Number of quantitative rules for each of 16 categories.

The optimum DT model adopted the top 2,500 features in the mRMR feature list. Accordingly, DT was executed on the dataset containing all 4,986 proteins, thereby constructing a big tree. From this tree, 760 quantitative rules were extracted, which are provided in Supplementary Table S4. Each of 16 categories was assigned some rules. Figure 5 shows the number of rules for each of 16 categories. Some categories (e.g., Class 1: Biological membrane, Class 3: Cytoplasm) received more than 100 rules, whereas there were only three rules for Class 8: Flagellum or cilium. In *Quantitative Rules That Contribute to Subcellular Localization Prediction*, several rules would be analyzed.

### 3.4 Comparison of the Classic Model

The pseudo-amino acid composition (PseAAC) (Chou, 2001) is a classic protein encoding scheme and has been widely adopted to build models for identification of protein subcellular locations (Cai and Chou, 2003; Pan et al., 2003; Lin et al., 2008; Shi et al., 2008; Liu et al., 2010). Here, we used such scheme to encode each protein mentioned in *Data* and further build models for the comparison of models proposed in this study.

**FIGURE 6:**
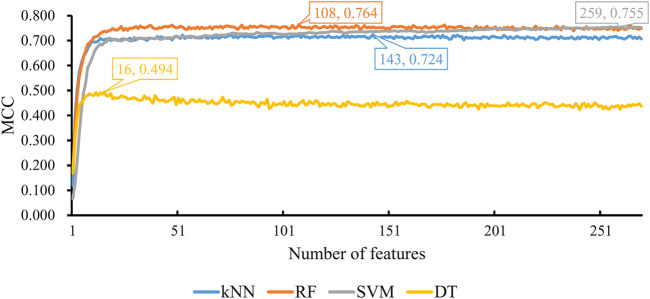
IFS with four classification algorithms on mRMR feature list of PseAAC features. The highest MCC values obtained by four classification algorithms are 0.724, 0.764, 0.755, and 0.494, respectively.

Five physicochemical and biochemical properties of amino acids were employed to generate features, including codon diversity, electrostatic charge, molecular volume, polarity and secondary structure. The weight factor was set to 0.15 and Lambda parameter was set to 50. From each physicochemical and biochemical property, 50 features were extracted. Thus, 250 (50✕5) features were obtained for each protein. Furthermore, 20 amino acid composition features were also employed. Accordingly, each protein was represented by 270 (250 + 20) features. These features were directly analyzed by mRMR method, resulting in a feature list. Such list was fed into the IFS method. Likewise, four classification algorithms: kNN, RF, SVM, and DT, were also tried in the IFS method. For each classification algorithm, MCC values obtained on all possible feature subsets are illustrated in Figure 6. It can be observed that the highest MCC values for four classification algorithms were 0.724, 0.764, 0.755, and 0.494, respectively, which are also listed in Table 3. The corresponding ACC values are also listed in this table. Compared with ACC and MCC values obtained by models using network and functional features (Table 2), with the same classification algorithm, our models were superior to models with PseAAC features. It was suggested that network and functional features were more efficient than PseAAC features for identification of protein subcellular locations. These features provided new directions for building more efficient protein subcellular location prediction models.

**TABLE 3:** Performance of the optimum models using PseAAC features.

Classification algorithm	Number of features	Overall accuracy	MCC
k-nearest neighbor	143	0.757	0.724
Random forest	108	0.803	0.764
Support vector machine	259	0.794	0.755
Decision tree	16	0.559	0.494

## 4 Discussion

A group of effective proteins that may directly contribute to the identification and clustering of different subcellular localizations is screened by using some machine learning models. According to recent publications, the top optimal features have already been validated to contribute to the subcellular localization, validating the efficacy and the accuracy of our predictions. The detailed analyses and discussion can be seen below.

### 4.1 Features From Proteins That Contribute to Subcellular Localization Prediction

The first feature protein is **SUMO2** (ENSP00000405965). According to recent publications, this protein is a member of the small ubiquitin-like modifier family and contributes to ubiquitin-mediated post-translational modification system by acting as a signal for proteasomal degradation (Hecker et al., 2006; Tammsalu et al., 2014). In 2013, a research on testis functions confirmed that SUMO2 is specifically located in the nucleus region of the cell and is mediated by retinoic acid (Zhu et al., 2010). Therefore, this protein is a potential feature for specific subcellular regions.

The following feature protein is NDUFS3 (ENSP00000263774). As a specific iron–sulfur protein component of the mitochondrial NADH, this protein participates in the electron transport in the mitochondrion and contributes to energy-associated metabolisms in living cells (Benit et al., 2004). This protein is located in the mitochondrial and the nucleus regions (Vogel et al., 2007b; Taurino et al., 2012). Specifically, most of this protein is directly located and functions in the inner mitochondrion membrane (Benit et al., 2004; Vogel et al., 2007a).

GRK3, the next predicted feature protein (ENSP00000317578), acts as a beta-adrenergic receptor kinase, contributes to the GPCR signaling pathway (Antony et al., 2009; Kumari et al., 2016), and participates in the CCR5 pathway in macrophages (Vroon et al., 2004; Balabanian et al., 2008). In general cells, GRK3 does not have a specific localization pattern. However, in macrophages, this protein merges with CXCR4 to form specific complexes in the cellular membrane system (Wang et al., 2001). Therefore, in these functional cells, our candidate protein GRK3 has a specific spatial distribution pattern and may contribute to the identification of the biological membrane region, validating the efficacy and the accuracy of our prediction.

BRIX1 (ENSP00000338862) is the specific regulator in the biogenesis of the 60S ribosomal subunit and is predicted to contribute to subcellular localization (Fromont-Racine et al., 2003; Strunk and Karbstein, 2009). According to recent publications, this protein is mostly located inside the nucleus and regulates ribosome biosynthesis (Zieve and Penman, 1976; Nguyen et al., 1998). According to the Human Protein Atlas (HPA), this protein is identified in the cytoplasm, but most of the protein is still located and functions in the nucleus, validating that the specific subcellular localization subgrouping is dependent on this protein (Pontén et al., 2008).

MDH2 (ENSP00000327070) contributes to the catalyzation of the reversible oxidation of malate to oxaloacetate and is predicted to help in the identification of a certain subcellular region (Pines et al., 1997; Shi and Gibson, 2011). According to HPA (Pontén et al., 2008), like NDUFS3, this protein is mostly identified in the mitochondrion. Recent publications also confirm that this protein can be identified in multiple intracellular organelles but is actually enriched in the mitochondria system (Lo et al., 2015) especially the mitochondria-associated ER membranes (Guardia-Laguarta et al., 2014; Lo et al., 2015). Moreover, this protein acts as a potential subcellular signature and corresponds with our prediction.

The H3-3B (ENSP00000254810) in our prediction list is the basic nuclear protein that contributes to the maintenance of the chromosomal fiber in eukaryotes (Frey et al., 2014). Therefore, this protein is definitely located in the nucleus region, thereby indicating subcellular localization. Similar with BRIX1, the protein NHP2 (ENSP00000274606) is a specific protein required for ribosome biogenesis (Vulliamy et al., 2008; Fumagalli et al., 2009) and telomere maintenance (Wong and Collins, 2003; Vulliamy et al., 2008). Therefore, this protein is also identified in the cytoplasm and the nucleus. This protein has potential to act as a subcellular localization signature because most of it is located in the nucleus (Pontén et al., 2008). Other feature proteins, e.g., CYC1 (ENSP00000317159) (Chen et al., 1994) and H2AZ2 (ENSP00000308405) (Eskandarian, 2013), have specific distribution patterns inside the cell, cytoplasm, and nucleus according to recent publications.

Overall, the feature proteins we analyzed have already been validated to contribute to the subcellular localization, validating the efficacy and the accuracy of our prediction. Thus, our newly presented computational method may be an effective tool for the prediction of subcellular localizations.

### 4.2 Features From Functions That Contribute to Subcellular Localization Prediction

The functional enrichment analysis is performed, and a group of effective GO (Consortium, 2015) and KEGG terms (Kanehisa, 2002) is screened to describe the core biological functions related to subcellular localization and further show the functional distribution pattern of feature proteins.

The top four GO terms in our prediction list describe specific subcellular localization or effective structures contributing to the distinction of different subcellular localization. These terms include GO:0070013 (describes the intracellular organelle lumen), GO:0031975 (describes the specific envelope structures in cells), GO:0031090 (describes the organelle membrane), and GO:0005887 (describes the integral component of the plasma membrane).

For example, the intracellular organelle lumen is a specific part of effective organelles, such as mitochondrion, peroxisomes, and Golgi apparatus (Lorenz et al., 2006a; Lorenz et al., 2006b; Masyuk et al., 2006), distinguishing perticular subcellular localization from the other ones. Therefore, GO:0070013 can contribute to subcellular localization. For GO:0031975, the envelope is a multilayered structure connected to the cell membrane or other membrane systems (Peabody et al., 2016). Therefore, this GO term is functionally correlated with the cell membrane and with various organelles with membrane-like mitochondrion and Golgi apparatus (Graham et al., 1991; Finnegan et al., 2001; Peabody et al., 2016). Other subcellular localization prediction algorithms also consider this term as a specific parameter for classification (Peabody et al., 2016). Similarly, GO:0031090 and GO:0005887 describe a part of the membrane system in cells.

### 4.3 Quantitative Rules That Contribute to Subcellular Localization Prediction

Apart from the qualitative analysis on specific GO or KEGG terms, a group of quantitative rules are established for the identification of different subcellular localizations. According to recent publications, these rules contribute to subcellular localization, thereby validating the efficacy and the accuracy of our prediction. Here, 16 typical rules referring to 16 clusters are chosen for detailed analyses.

The first rule is to identify the biological membrane subcellular localization (Class 1). According to the quantitative rules, the first parameter is GO:0031224. According to our prediction, the proteins enriched in this cellular component positively contribute to the biological membrane. Considering that GO:0031224 describes the intrinsic component of membrane, this GO term is the first parameter to identify the proteins associated with the biological membrane, validating our prediction. Similarly, GO:0005886 describes the plasma membrane and may positively contribute to the identification of such subcellular localization. Some terms negatively participate in this identification. For instance, the nuclear lumen described by GO:0031981 located inside the nucleus is in our prediction list.

For the rules that contribute to the identification of cell periphery subcellular localization (Class 2), GO:0031224 is in this predictive parameter list. The specific GO term GO:0007043 highly enriches proteins associated with the identification of cell periphery subcellular localization. According to the GO annotation, this GO term describes the cell–cell junction assembly, which definitely occurs in the periphery subcellular regions (Setzer et al., 2004; Dawson et al., 2012), validating the efficacy and the accuracy of our prediction.

The third rule focuses on the identification of cytoplasm (Class 3). Specifically, wound healing (GO:0042060) is identified as a specific positive enrichment marker for this rule. The cytoplasm plays an essential role for wound healing (Jeon and Jeon, 1975). Therefore, the proteins that are located at the cytoplasm can be identified by a specific biological process (Jeon and Jeon, 1975; Gabbiani et al., 1978), such as wound healing.

Similar with that of the cytoplasm, a group of rules for the identification of cytoplasmic vesicle (Class 4) are identified. Among the rule parameters, the specific GO term GO:0070727 that describes the cellular macromolecule localization (Franklin and Baltimore, 1962) is a key feature that contributes to the identification of the cytoplasmic vesicle. According to recent publications, the cytoplasmic vesicle is a major transporter of macromolecules during synthesis and functioning (Franklin and Baltimore, 1962). Therefore, this GO term is a distinctive parameter for the sublocation of the cytoplasmic vesicle.

Furthermore, some specific rules are identified for endosome (Class 6), extracellular space or cell surface (Class 7), and flagellum or cilium (Class 8). Apart from some general GO terms, such as GO:0031224, the GO:1902115 is a specific parameter for the identification of endosome. Describing the assembly of effective intracellular organelles, this GO term contributes to the identification of endosome subcellular localization due to the tight correlation between endosome and organelle assembly (Kjeken et al., 2004; Kloer et al., 2010). For the identification of the extracellular space or the cell surface, apart from a series of GO terms like other predictive rules, the specific protein SDAD1 is obtained for the prediction of subcellular localization on the extracellular space or the cell surface. According to recent publications, this protein is located mostly inside the nucleus (Zeng et al., 2017) but not outside or on the biomembrane system. As for flagellum or cilium (Class 8), a specific parameter called GO:2000816 is positively correlated with the identification of this subcellular localization. This GO term describes the negative regulation of mitotic sister chromatid separation. Considering that mitotic separation is one of the major biological functions of the centriole–flagellum system (Wilson, 1969; Bettencourt-Dias et al., 2005), this parameter (biological process) is correlated with our predicted subcellular localization to a certain extent and definitely contributes to the identification of this cellular structure, thereby validating our predictions.

In addition, specific organelles, such as endoplasmic reticulum localization (Class 5), Golgi apparatus (Class 9), and mitochondrion (Class 11), can be identified and located by specific quantitative rules. The specific parameter GO:0005789 contributes to the localization of the endoplasmic reticulum. The GO:0005789 describes the endoplasmic reticulum membrane, validating the efficacy and the accuracy of our prediction. For the localization of Golgi apparatus, the specific parameter has00601 describing the glycosphingolipid biosynthesis is identified. Considering that glycosphingolipid biosynthesis is a typical biological process happening in the Golgi apparatus (Burger et al., 1996; Butters et al., 2000), this function is predicted as a quantitative parameter for the identification of Golgi apparatus subcellular localization. The mitochondrion is the next predicted subcellular localization with typical predictive parameters (such as GO:0031975), and the envelope is analyzed above (Peabody et al., 2016). This GO term is functionally correlated with the mitochondrion (Graham et al., 1991; Finnegan et al., 2001; Peabody et al., 2016), confirming our prediction.

Furthermore, the cell nucleus-associated locations, such as nuclear periphery (Class 12), nucleolus (Class 13), and nucleus (Class 14), can be quantitatively identified by our rules. For class 11, nuclear periphery regions are identified. Apart from the typical parameters, such as GO:0031981 and GO:0005654, the typical protein ENSP00000345895 or NUP50 is identified. According to recent publications, this protein is enriched in the periphery regions of the nucleus (Hajeri et al., 2010; Vaquerizas et al., 2010), thereby positively corresponding with our prediction. For the nucleolus, the specific biological process RNA surveillance (GO:0071025) is enriched in such rules as an effective parameter. Considering that RNA surveillance does occur in this region (Hernandez-Verdun et al., 2010), this GO term is a functional predictive parameter, validating the efficacy and the accuracy of our prediction. Similar with the nucleolus, the nucleus has its specific “biomarkers” in these quantitative rules. GO:0045596 describes the negative regulation of cell differentiation and is positively enriched in these rules. Considering that the physical plasticity of nucleus is quite important for cell differentiation (Pajerowski et al., 2007), this GO term is a positive parameter for subcellular localization, validating the efficacy and the accuracy of our prediction.

Three effective subcellular regions, namely, microtubule cytoskeleton (Class 10), peroxisome (Class 15), and vacuole (Class 16) remain. For the identification of microtubule cytoskeleton, the typical GO term GO:0044450 describes the obsolete microtubule organizing center part and is functionally and positively correlated with the microtubule system. Therefore, the predicted quantitative rules may be effective for the identification of the microtubule cytoskeleton’s subcellular localization. Peroxisome identification requires the specific quantitative parameter GO:0031903, which describes the microbody membrane. According to recent publications, peroxisomes are major functional components of the microbody. Thus, this GO term is an effective parameter (Fahimi, 1969; Tolbert and Essner, 1981). The last subcellular localization is the vacuole. Similar with the peroxisomes’ rules, a specific GO term describing only the vacuolar lumen, a part of the vacuole, is identified, thereby validating our prediction.

## 5 Conclusion

We identified a group of feature proteins that effectively contributes to intracellular subcellular localization and screened a series of qualitative functional enrichment patterns (i.e., GO and KEGG terms), revealing the functional distribution patterns of these proteins that contribute to subcellular localization identification. Combining proteins and functional annotations, a series of quantitative prediction rules was built for further analysis. Several screened feature proteins, functional annotation terms (i.e., GO or KEGG terms), and parameters of quantitative rules have been validated by recent publications. This study can provide a computational model for effective subcellular localization prediction and lay a solid foundation for further experimental research in such fields. The data as well as the information of used programs and software are available at https://github.com/chenlei1982/subc_prediction.

## Data Availability

Publicly available datasets were analyzed in this study. This data can be found here: http://cn.expasy.org/
